# Parental marital relationship satisfaction is associated with glycemic outcomes in children with type 1 diabetes

**DOI:** 10.1007/s40200-022-01084-2

**Published:** 2022-07-29

**Authors:** Lindsey A. Loomba, Amy Hughes Lansing, Justine N. Cortez, Kearnan Welch, Joe N. Solowiejczyk, Simona Ghetti, Dennis M. Styne, Nicole S. Glaser

**Affiliations:** 1 Department of Pediatrics, Section of Endocrinology, University of California Davis Medical Center, 2516 Stockton Blvd., Suite 384, 95817-2208 Sacramento, CA USA; 2 Department of Psychological Science, University of Vermont, 05405 Burlington, VT USA; 3 Department of Internal Medicine, Division of Endocrinology, University of California Davis Medical Center, 95817 Sacramento, CA USA; 4 Department of Psychology, Center for Mind and Brain, University of California Davis, 95616 Davis, CA USA; 5 Children’s Hospital Los Angeles, 90095 Los Angeles, CA USA

**Keywords:** Type 1 diabetes, Children, Glycemic control, Family dynamics, Marital satisfaction

## Abstract

**Objectives:**

We hypothesized that glycemic outcomes in children with type 1 diabetes are linked to marital satisfaction of primary caregivers above and beyond parent neuroticism and child effortful control.

**Methods:**

We evaluated a cross-sectional sample of 73 married parent families with a child (ages 7–18 years) with type 1 diabetes of at least 2 years duration. We assessed marital relationship satisfaction, parent neuroticism, and child effortful control through the use of validated questionnaires. We used univariate comparisons and multivariable models to determine whether marital relationship satisfaction was associated with hemoglobin A1c [HbA1c] and whether this association persisted after adjusting for demographic factors and parent neuroticism/child effortful control.

**Results:**

In univariate analyses, HbA1c was associated with marital relationship satisfaction of the primary caregiver. In multivariable models adjusting for demographic factors, marital satisfaction remained associated with HbA1c, whereas none of the other factors tested (including family income and race/ethnicity) retained significance. In univariate analyses, child effortful control was also associated with HbA1c. When child effortful control was added to the multivariable model, marital satisfaction remained associated with HbA1c with similar coefficient and confidence intervals describing the relationship between marital satisfaction and hemoglobin A1c.

**Conclusions:**

Higher levels of marital satisfaction of the primary diabetes caregiver are associated with glycemic outcomes for children with type 1 diabetes. Interventions to improve spousal relationships may have downstream benefits that could include promoting more optimal child HbA1c levels.

## Introduction

Achieving targets for glycemic outcomes in children with type 1 diabetes (T1D) depends not only on access to state-of-the-art insulin regimens, diabetes technologies and education, but also on the abilities of the children and their families to determine insulin dosing, administer correct doses of insulin multiple times daily and regularly manage both hypo- and hyperglycemia. Instruction by diabetes educators is routinely provided, yet only a minority of children with diabetes in the United States achieve target glycemic outcomes (HbA1c < 7.0% (53mmol/mol)) [1, 2]. Previous studies suggest that neither knowledge deficits amongst children/caregivers nor physiological differences account for the sub-optimal glycemic outcomes [3, 4].

Research has previously identified multiple indicators of family structure that are associated with suboptimal glycemic outcomes in youth with T1D. For example, children in blended and single parent families are more like to experience poor glycemic outcomes [5–8], suggesting that marital family structure is a critical index of socioeconomic disadvantage that impacts T1D management. However, research has *not* yet examined the role of marital relationship satisfaction in explaining glycemic outcomes, and there is evidence from community samples that this is an important construct. In adolescent community samples, poor parental marital quality predicted worse physical and mental health [9, 10], while in younger children it has been linked to worse physical health [11]. This suggests that lower parental marital satisfaction may impact health behaviors and outcomes for children with type 1 diabetes.

In examining the association of martial relationship satisfaction with suboptimal glycemic outcomes, it is also critical to consider whether any association is unique from individual parental and child factors associated with both interpersonal, coping, and self-regulation skills as well as martial satisfaction. For example, both child effortful control and parent neuroticism are linked with sub-optimal glycemic levels and martial satisfaction. Lower child effortful control (an early temperament trait describing a child or adolescent’s ability to self-regulate emotions, cognition, and behaviors [12]) is associated with both lower parent marital relationship satisfaction in general [11, 13–15] and with suboptimal child diabetes self-management and glycemic outcomes in children with T1D [9, 16–19]. Greater parent neuroticism (the extent to which a person has a tendency to experience negative emotions, anxiety, and depression [20]) and neuroticism-related processes in parents of children with T1D are also associated with suboptimal glycemic outcomes [3, 9, 21–26] and neuroticism is associated with lower marital relationship satisfaction in community samples [27–29]. Given that both parent neuroticism and child effortful control are associated with marital relationship satisfaction *and* glycemic outcomes, it is critical to explore whether the association of parental marital satisfaction with hemoglobin A1c (HbA1c) is unique from, or already accounted for by, the association of parent neuroticism or child effortful control and HbA1c.

We undertook the current study to determine whether parent marital relationship quality, including parent-reported marital satisfaction and child-reported interparental conflict frequency, is associated with glycemic levels. First, we hypothesized that higher parent marital relationship quality and lower child interparental conflict ratings would be associated with more optimal glycemic levels above and beyond demographic factors. Second, we hypothesized that, given the centrality of the family system for diabetes management, parent marital relationship quality would be associated with more optimal glycemic levels above and beyond parent neuroticism and child effortful control. Additionally, based on findings from previous studies, we anticipated that children in married parent families would have more optimal glycemic levels compared to single parent families.

## Methods:

To determine whether parent marital relationship quality is associated with glycemic outcomes in children with established diabetes, we evaluated a cross-sectional sample of 73 children (ages 7–18 years) with type 1 diabetes from married parent families and their parents. 26 children from single-parent families were included to examine covariation between demographic factors and glycemic control and describe and compare glycemic control between groups. Participants were recruited through an academic hospital pediatric endocrinology clinic. The study was approved by the University of California, Davis Institutional Review Board (which serves as the local Ethics Committee for human research).

Participants completed validated questionnaires relating to marital relationship satisfaction, parental neuroticism and child effortful control (see below). Univariate analyses and multivariable models were used to determine if marital relationship satisfaction was associated with HbA1c and if any association persisted after adjustment for demographic factors, parental neuroticism and child effortful control.

### Participants

Families were eligible for participation if:

1. The child had type 1 diabetes of at least two years duration (to ensure that endogenous insulin production would not influence glycemic control).
2. The child was utilizing a basal-bolus (insulin pump or multiple daily injection) insulin regimen.
3. For married parent households, both parents stated willingness to complete the study assessments. (For single parent households, one parent completed the study assessments).

Families were excluded from the study if they were non-English or non-Spanish speaking (n = 1) or if the child or the caregivers had severe underlying psychiatric or medical conditions that could independently affect either glycemic outcomes or family dynamics (n = 2). Eligible participants were identified by reviewing medical records for inclusion and exclusion criteria. Eligible participants were approached in the diabetes clinic by a research coordinator who explained the study and obtained written informed consent from parents and assent from children. A gift card was provided as an enrollment incentive.

Married primary caregiver families were defined as any families in which the primary caregiver was married and the spouse had the potential to participate in daily diabetes care in the home, regardless of the status of the spouse as a biological or step-parent to the child with diabetes (n = 73). Children in single parent families and their primary caregivers were eligible to participate in assessments related to broader study aims but were not included in the primary marital satisfaction analyses in this manuscript (n = 26). For all family structures, a parent was defined as the child’s father or mother, and could include biologic parents, step-parents or adoptive parents.

Children enrolled in the study received routine comprehensive diabetes education provided at diagnosis from a multidisciplinary team, with followup visits typicaly occurring every three months. This did not differ from the clinical care routinely provided in the clinic.

### Data Collection

We recorded the child’s HbA1c at the time of study enrollment, and additionally recorded the child’s mean HbA1c level (average of all HbA1c levels collected as a part of routine clinical care) in the 12 months prior to study enrollment. HbA1c data in the 12 months prior to enrollment was obtained via search of the patient’s electronic medical record. HbA1c at the time of study enrollment was used as the primary measure of glycemic control, however, additional analyses were also performed using mean HbA1c over the year prior to study enrollment as the outcome measure. Mean HbA1c during the prior year was included to assess the extent to which transient events (e.g. illnesses, travel, changes in diabetes care equipment) temporarily influence HbA1c and may cause the single point HbA1c to be poorly reflective of the child’s usual glycemic levels. We additionally recorded duration of diabetes and age at diagnosis. We recorded the relationship of the primary diabetes caregiver/s to the child, marital status of the child’s primary caregiver, household income, race/ethnicity and educational attainment of the caregiver/s from a demographic questionnaire. The child’s current grade point average (GPA) was included as a measure of overall academic functioning [30, 31] and was estimated from caregiver reports using a scale of “all A’s” (4.0), “A’s and B’s”(3.5), “all B’s” (3.0) and so on.

Study questionnaires were offered in both English and Spanish and were administered by a single trained research assistant in the clinic setting. All participants opted to use English language questionnaires. Caregivers completed the questionnaires in a single session averaging 30–60 min; spouses did not have access to each other’s questionnaire responses. When necessary due to inadequate reading skills, the research assistant read and explained questions to the child. Children assented to study participation, and a trained social worker was available should questionnaire completion provoke psychological distress. The following measures were used:

**Relationship Satisfaction Scale –** A subset of the Investment Model Scale, [32, 33] a validated instrument designed to measure four relationship domains, including satisfaction level. The scale was completed by both the primary caregiver and spouse and comprises five items measuring the degree to which the relationship gratifies the individual’s need for intimacy, companionship, sexuality, security and emotional involvement. Items are rated on a 4-point scale (1 = don’t agree at all, 2 = agree slightly, 3 = agree moderately, 4 = agree completely). Higher mean scores indicate greater relationship satisfaction. Good reliability for this scale was evidenced in the present sample (primary caregiver α = 0.96; spouse α = 0.87).

**Child Perception of Interparental Conflict Scale- Short Revised (CPIC-SR)** [34, 35]. Child’s assessment of conflict between parents was evaluated using this 25 item measure, which is validated for use in all ages of children enrolled in our study. All children ages 7–18 years reported on the intensity, content (perception of child that he/she is involved or blamed in the conflict), duration and resolution of conflict between parents. Questions (i.e. “When your parents have an argument or disagreement, they usually work it out”) were rated on a 5-point scale (1 = strongly disagree to 5 = strongly agree). Only the frequency of conflict scale was used in this analysis. Sufficient reliability for this scale was evidenced in the present sample (α = 0.78).

**NEO–Five Factor Inventory (NEO-FFI-R), Neuroticism Factor** [20, 36]. This 60 item assessment of the five factor model of personality was administered to all parents. Only the neuroticism factor score was used in this study, which was generated from responses to 12 of the questions. Questions (e.g. “At times I have felt bitter and resentful.”) are rated using a 5-point scale (1 = strongly disagree to 5 = strongly agree). Good reliability for this scale was evidenced in the present sample (primary caregiver α = 0.80; spouse α = 0.90).

**Child Effortful Control.** Child effortful control was assessed using the 157 item Temperament in Middle Childhood Questionnaire (TMCQ) or the 65 item Early Adolescent Temperament Questionnaire (EATQ), Revised Short Form [12, 37–41]. The TMCQ was used to assess parent reports of child effortful control for 7–10 year old children, whereas the EATQ was used for parent reports of children ages 11–15 years *and* for child self-reports in children ages 9–15 years. Items for both measures are rated on a 5-point scale from 1 (“almost always untrue”) to 5 (“almost always true”). Sample questions include “My child has an easy time waiting to open a present.” For both the TMCQ and EATQ, the effortful control score is calculated as a superscore of component subscales [12, 37–41]. For this study parent and child reports were converged together into a single temperament superscore for effortful control. Mean scores for both parents’ assessments were used for two parent families; single parent scores were used in single parent families [42, 43]. Sufficient to good reliability for each reporter on this scale and across reporters was evidenced in the present sample (EATQ by reporter teen α = 0.72, parent 1 α = 0.83, parent 2 α = 0.65; TMCQ by reporter primary caregiver α = 0.85, spouse α = 0.66; across all three reporters and EATQ and TMCQ α = 0.82).

Due to lack of validated questionnaires for their age group, 16–17 year olds did not have temperament assessed in this study (n = 24). Due to low enrollment and to avoid non-continuous data, 18 year olds (n = 6) were also excluded from the temperament analyses.

### Statistical Analyses

First, to determine representativeness of the study population, we compared demographic and clinical data for our sample to data for our diabetes clinic as a whole. Second, we conducted univariate analyses of demographic variables and marital relationship satisfaction with HbA1c. Univariate associations between demographic and psychosocial variables and glycemic outcomes were evaluated using Pearson’s correlation coefficients. Student’s t-test was used to assess differences in HbA1c between groups defined by dichotomous variables. Analysis of variance was used to determine the effects of multiple category variables (household income, parental education level) on glycemic outcomes.

Third, we conducted multiple forced linear regression analyses to examine whether marital status and parental marital relationship satisfaction were associated with glycemic control above and beyond demographic factors. All demographic factors with significant or near-significant (p < 0.10) univariate associations were entered into the regression models. These included income, race/ethnicity, educational level of the child’s father and the child’s grade point average (GPA). Because frequencies of type 1 diabetes are highest in white populations, the number of children in minority racial/ethnic groups was small. Minority racial/ethnic groups were therefore combined into one group in the analyses. Because we hypothesized that predictive variables would have threshold effects and for ease of interpretation, all predictive variables were entered into the regression analyses in dichotomous form. For these analyses, low annual income was defined as $75,000 or less. This value was selected based on income averages for families in California reported by the U.S. Department of Housing and Urban Development (HUD). A GPA of 3.5 or higher (50th percentile for the sample) was used to define high achieving students. Father’s educational level was defined as low if it did not include at least one year in college.

High marital satisfaction was defined a priori as a score of 3.0 or higher, and was analyzed as a dichotomous variable due to anticipated threshold effects. High marital satisfaction included individuals who reported moderate to complete agreement (average rating of 3 or 4) with their relationship matching the item descriptions of good relationship satisfaction. Low marital satisfaction included individuals who reported slight or no agreement (average rating of 1 or 2) with their relationship matching the item descriptions of good relationship satisfaction.

Fourth, we conducted univariate analyses of the association of child effortful control and, where caregiver marital satisfaction was associated with HbA1c, parent neuroticism with glycemic outcomes. Last, for any significant parent neuroticism or child effortful control association with HbA1c, we conducted additional forced linear regression analyses. These regressions examined whether previously significant marital status and parental marital relationship satisfaction were associated with glycemic control above and beyond demographic factors and parent neuroticism or child effortful control.

## Results

147 families presenting to the pediatric diabetes clinic during a two year enrollment period met enrollment criteria for the cross-sectional sample, and 102 of these families (69%) were enrolled. 99 families completed the study and three dropped out prior to completion (all as a result of failure to complete surveys due to time constraints). Families that declined enrollment cited reasons including time constraints (n = 18), lack of interest in study (n = 17), lack of a parent or legal guardian present at the visit (n = 7), and discomfort completing the questionnaires (n = 2). Characteristics of the study participants were similar to those of our clinic population overall, including mean age (13.3 ± 3.1 vs. 12.8 ± 4.3 years, p = 0.35), sex (54% vs. 51% female, p = 0.71), HbA1c at the time of study enrollment (8.8 ± 1.4 vs. 8.6 ± 1.5% [74 ± 17.5 vs. 70 ± 16.4mmol/mol], p = 0.13), and duration of diabetes (6.7 ± 3.4 vs. 6.5 ± 3.7 years, p = 0.43). Mean HbA1c during the year prior to study enrollment (8.8 ± 1.5%) was similar to HbA1c at the time of enrollment. 73 enrolled families with married parents comprised the main study group. Data from 26 additional families with single parents were analyzed to examine covariation between demographic factors and glycemic control. Demographic characteristics of the study population are described in Table 1.

**Table 1:** Demographic Characteristics of Study Population (n = 99)

Age at enrollment (years)	13.3 (3.1)*
**Age at diagnosis (years)**	6.7 (3.6)
**Duration of diabetes (years)**	6.7 (3.4)
**HbA1c (%)** **At time of study enrollment** **Average HbA1c over year prior to enrollment** ^**+**^	8.8 (1.4) 8.8 (1.5)
**Gender - n (%) female**	54 (54%)
**Race/Ethnicity** ^**‡**^ **White** **African-American** **Hispanic** **Another**	74 (75%) 6 (6%) 10 (10%) 9 (9%)
**Marital status of parents** **n (%) married**	73 (74%)
**Child’s diabetes caregiver/s** **Mother alone** **Father alone** **Both parents** **Mother plus grandparent/s** **Grandparent/s alone** **Child completely independent**	37 (37%) 4 (4%) 45 (45%) 7 (7%) 2 (2%) 4 (4%)
**Child’s primary caregiver for purposes of survey responses (self-selected by families)** **Child’s mother** **Child’s father**	94 (95%) 5 (5%)
**Household annual income** **<$75,000/year** **>$75,000/year**	58 (59%) 40 (41%)
**Mother’s educational level** ^**§**^ **High school or lower** **1 + years of college** **4-year college or post-graduate**	22 (22%) 50 (51%) 23 (23%)
**Father’s educational level** **High school or lower** **1 + years of college** **4-year college or post-graduate**	26 (26%) 37 (37%) 31 (31%)
**Child’s GPA**	3.07 (0.88)

* Values presented as mean (SD)

*+ average number of HbA1c measurements included in mean HbA1c = 3.5 (SD 1.1)*

‡ “*Another” includes Asian, Native American, Pacific Islander*

*§ Educational levels do not total 100% due to missing data*

### Associations of parent relationship satisfaction and glycemic outcomes

First, covariation between demographic factors and glycemic control was examined in the full sample (n = 99) (Table 2). Neither the child’s age, gender, duration of diabetes nor age at diagnosis of diabetes was associated with HbA1c. There was a significant negative correlation between HbA1c and the child’s GPA. Lower educational level of the child’s father and lower household income were both associated with higher HbA1c. White race showed a trend toward association with lower HbA1c in the primary analyses (p = 0.06) and was associated with lower mean HbA1c over the prior 12 months in secondary analyses (p = 0.005).

**Table 2:** Relationship of HbA1c to demographic variables

Continuous Variables	Correlation coefficient (p-value)		
**Age (years)**	0.10 (p = 0.34)		
**Age at Diagnosis of Diabetes**	-0.04 (p = 0.70)		
**Duration of diabetes**	0.14 (p = 0.15)		
**Child’s school GPA**	-0.34 (p < 0.001)		
**Categorical Variables**	**Mean HbA1c (SD) in each group**	**p-value for comparison of groups**	
**Gender** Male Female	9.0 (1.7) 8.9 (1.6)		p = 0.62
**Income** <$75,000/year >$75,000/year	9.4 (1.7) 8.2 (1.2)		p < 0.001
**Mother’s education level**: High school or less 1 + years college 4-year college or post-graduate	8.7 (1.4) 9.2 (1.8) 8.3 (1.5)		p = 0.36
**Father’s education level**: High school or less 1 + years college 4-year college or post-graduate	9.3 (1.7) 8.8 (1.2) 8.7 (1.9)		p = 0.03
**Race/ethnicity** White Person of color or Hispanic ethnicity	8.8 (1.4) 9.5 (2.0)		p = 0.06

**Fig. 1:**
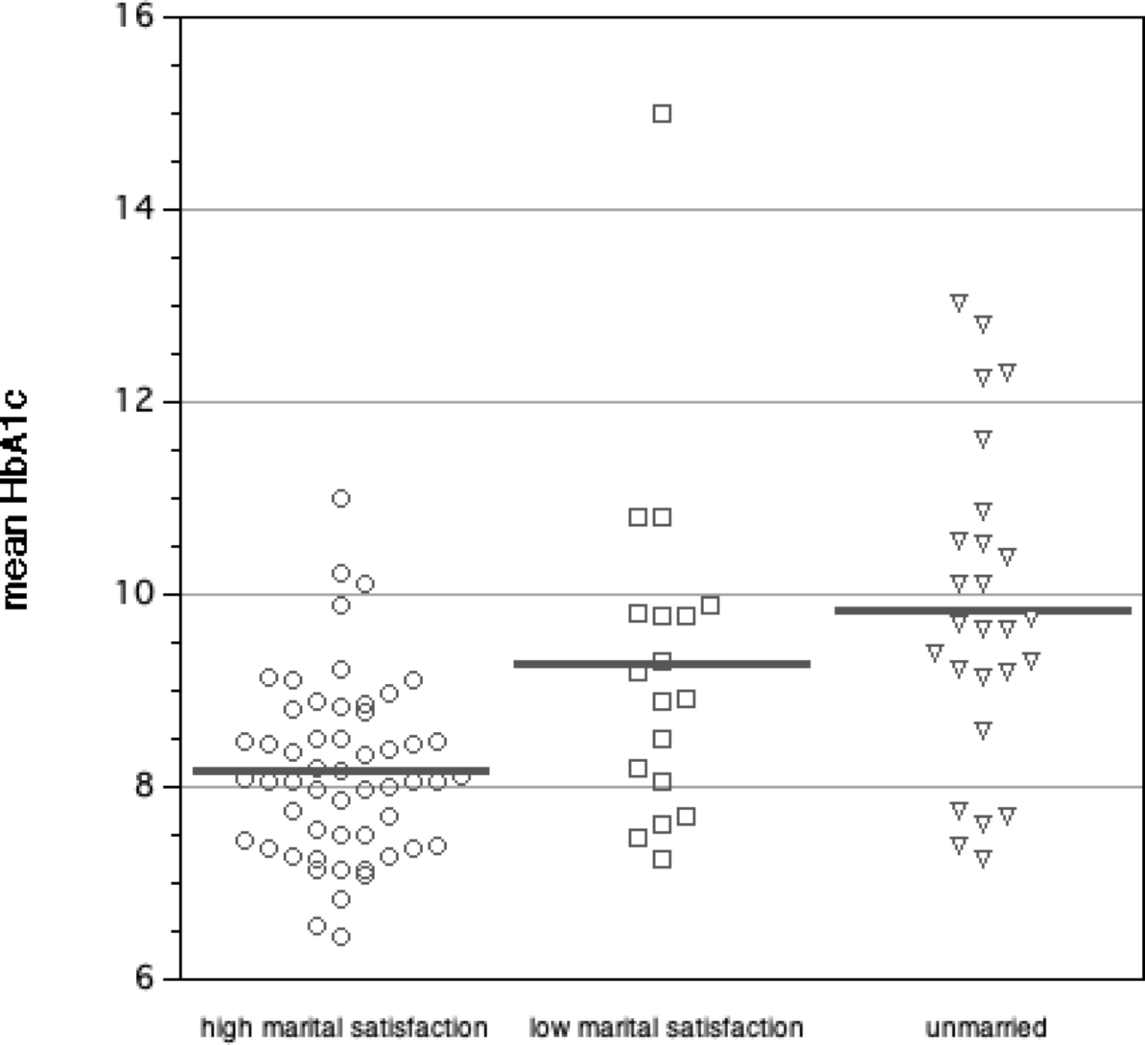
HbA1c values according to marital satisfaction and marital status. *High and low marital satisfaction shown for child’s primary diabetes caregiver. Mean HbA1c values = 8.1 (high marital satisfaction), 9.3 (low marital satisfaction) and 9.8 (unmarried); p = 0.001 high vs. low marital satisfaction, p < 0.001 married vs. unmarried*

Second, univariate covariation between measures of parent relationship satisfaction and glycemic outcomes were examined (Table 3). Children’s HbA1c was lower in families with married parents compared to those with non-married parents. For families with married parents, high marital relationship satisfaction of the child’s primary caregiver was associated with more optimal glycemic levels (8.3 ± 1.1% vs. 9.4 ± 1.8% (p = 0.004)). Similar results were found when comparing mean HbA1c over the preceding year between families with high versus low marital relationship satisfaction (8.1 ± 0.9 vs. 9.3 ± 1.8% (p = 0.001) (Fig. 1). The child’s rating of lower frequency of interparental conflict was not associated with glycemic levels in the primary analyses (correlation coefficient 0.17, p = 0.09) but was associated in subanalyses utilizing mean HbA1c over the prior year (correlation coefficient 0.26, p = 0.01).

**Table 3:** Associations of marital status and marital relationship satisfaction with HbA1c

Categorical Variables	Mean HbA1c (SD) in each group	p-value
**Marital status (n = 99)** Married (n = 73) Non-married (n = 26)	8.6 (1.4) 9.8 (1.9)	p = 0.002
**Relationship satisfaction** ^*^ **of primary caregiver, married parents only (n = 70)** ^*+*^ High Low	8.3 (1.1) 9.4 (1.8)	p = 0.004
**Relationship satisfaction of spouse, married parents only, (n = 35)** ^‡*§*^ High Low	8.1 (1.3) 8.8 (1.1)	p = 0.12

* A score of 3.0 or higher on the relationship satisfaction questionnaire was used to define high marital relationship satisfaction

^*+*^ *Primary caregiver relationship to child: mother (n = 69), father (n = 1); values do not total 73 due to missing data*

^‡^ *Spouse relationship to child: mother (n = 1), father (n = 34)*

^*§*^ *Primary caregiver and spouse totals are not equivalent due to missing data from spouse*

Third, using a multivariable model, we determined whether marital status and primary caregiver marital relationship satisfaction retained an association with glycemic outcomes after adjusting for other factors that were previously determined in univariate analyses to influence HbA1c. These included family income, race/ethnicity, academic performance (child’s GPA) and educational level of the child’s father. In the multivariable model, relationship satisfaction of the primary caregiver continued to be associated with mean HbA1c after adjusting for the effects of the other variables of interest (Table 4a). Notably, in this model only relationship satisfaction retained an association with HbA1c, suggesting that the effect of the quality of the parents’ relationship was associated with glycemic control above and beyond those of the other variables. Marital status also retained a significant unique association with HbA1c after adjusting for the effects of GPA, race/ethnicity, father’s educational level and household income (Table 4b).

**Table 4:** **a. Multivariable analysis of factors associated with HbA1c in married parents (n = 68)** ^*+*^ **– Demographic Factors and Marital Satisfaction**

Variable	Coefficient (95% confidence interval)	p-value
**Low household annual income**	0.35 (-0.35, 1.06)	0.32
**White ethnicity**	-0.16 (-0.91, 0.59)	0.66
**High GPA**	-0.51 (-1.18, 0.15)	0.13
**Father’s educational level**	0.28 (-0.53, 1.09)	0.49
**High marital relationship satisfaction**	-1.09 (-1.81, -0.38)	0.003*

**Table 4:** **b. Multivariable analysis of factors associated with HbA1c – Demographic Factors and Marital Status (n = 96)** ^*+*^

Variable	Coefficient (95% confidence interval)	p-value
**Low household annual income**	0.62 (-0.09, 1.32)	0.09
**White ethnicity**	-0.52 (-1.22, 0.17)	0.14
**High GPA**	-0.61 (-1.24, 0.03)	0.06
**Father’s educational level**	0.002 (-0.71, 0.72)	0.99
**Married parents**	-1.03 (-1.77, -0.29)	0.007

*Description of variables: Household annual income below $75,000, GPA above 3.5, marital relationship satisfaction score of primary caregiver above 3.0, father’s educational level of less than 1 + year of college (or lower)*

*p = 0.014 in sub-analysis utilizing mean HbA1c over the year prior to study enrollment

^*+*^*Totals do not equal 70 (*Table 4a*) and 99 (*Table 4b*) due to missing data*

### Associations of parent neuroticism and child effortful control and glycemic outcomes

Next, relationships between parent neuroticism, child effortful control, glycemic control and marriage satisfaction were examined in children ages 7–15 years. In univariate analyses, mean primary caregiver neuroticism (32.2 ± 7.02) was not associated with HbA1c (correlation coefficient 0.10, p = 0.33), although greater primary caregiver neuroticism was associated with lower reports of primary caregiver marital satisfaction (correlation coefficient − 0.48, p < 0.0001). Higher child effortful control was associated with lower HbA1c (correlation coefficient − 0.29, p = 0.01, but not with primary caregiver marital satisfaction (correlation coefficient 0.15, p = 0.30). In multivariable models that adjusted for child effortful control, GPA, income and race/ethnicity, marital status continued to be associated with HbA1c (p = 0.002, Table 5a). In married parent families, primary caregiver relationship satisfaction also continued to be associated with HbA1c in the multivariable model (p = 0.002, Table 5b). Child effortful control did not retain a significant association with HbA1c in these models.

**Table 5:** **a. Multivariable Model of Factors Associated with HbA1c – Demographic Factors, Child Effortful Control and Marital Status (n = 96)** ^*+*^

Variable	Coefficient (95% confidence interval)	p-value
**Low household annual income**	0.52 (-0.31, 1.35)	0.22
**White ethnicity**	-0.85 (-1.63, -0.08)	0.03
**High GPA**	-0.53 (-1.28, 0.21)	0.16
**Child effortful control**	-0.51 (-1.35, 0.33)	0.23
**Father’s educational level**	0.07 (-0.79, 0.94)	0.86
**Married parents**	-1.37 (-2.20, -0.53)	0.002

**Table 5:** **b. Multivariable Model of Factors Associated with HbA1c – Demographic Factors, Child Effortful Control and Marital Relationship Satisfaction (n = 51)** ^*+*^

Variable	Coefficient (95% confidence interval)	p-value
**Low household annual income**	0.09 (-0.76, 0.94)	0.84
**White ethnicity**	-0.42 (-1.24, 0.39))	0.30
**High GPA**	-0.2 (-1.22, 0.38)	0.30
**Child effortful control**	0.14 (-0.79, 1.08)	0.76
**Father’s educational level**	0.49 (-0.52, 1.49)	0.33
**High marital relationship satisfaction**	-1.28 (-2.08, -0.48)	0.002

*Description of variables: Household annual income below $75,000, GPA above 3.5, child effortful control – coefficient represents 1 unit increase in effortful control score, Marital Relationship Satisfaction score of 3.0 or higher, father’s educational level of high school only (or lower)*

^*+*^*Totals do not equal 99 (*Table 5a*) and 73 (*Table 5b*) due to missing data*

## Discussion

In the current study, we demonstrate that glycemic outcomes in children with type 1 diabetes are associated with the primary caregiver’s satisfaction in their relationship with a spouse, with a mean HbA1c difference of 1.2% in families reporting high compared to low marital relationship satisfaction. Associations between marital relationship satisfaction and HbA1c persisted even after controlling for demographic factors that influence HbA1c and after controlling for child effortful control. Consistent with other studies [5–7], our study also found that parental marital status was associated with child glycemic control.

To our knowledge, our study is the first to document an association between parental marital relationship satisfaction and glycemic outcomes in the child. One previous study in Norway investigated associations between parental relationship quality and HbA1c in children 1 to 15 years old [44]. That study found no significant associations between relationship quality and HbA1c, however, the study population differed in several ways from the current study. The study involved younger children (including infants and toddlers) with diabetes duration of 3 months or longer. This group would have included children still in the honeymoon phase where HbA1c is substantially influenced by endogenous insulin production. Furthermore, cultural norms and involvement of extended family members in diabetes care may differ between Norwegian and U.S. populations.

Our data add to other literature demonstrating that parental well-being is strongly associated with glycemic control in children with type 1 diabetes [23, 24]. In a widely recognized parenting model, parents’ personal psychological resources, child characteristics and contextual sources of stress and support (including the marital relationship) are specified as domains that influence parenting [45]. Our findings raise the possibility that a supportive and satisfying marital relationship might improve glycemic outcomes by enhancing the quality of co-parenting for diabetes related-tasks, reducing stress and family conflict, and improving dyadic coping between parents and children, as well as child self-regulatory skills that are critical to more independent self-management [46–49]. Further, the novel finding of a strong association between parental marital relationship satisfaction and glycemic outcomes in the child also suggests that interventions to improve parental relationships might be explored as a means of improving outcomes for children with type 1 diabetes and suboptimal glycemic levels.

As in other studies [9, 16–19, 50], we found that child effortful control was significantly associated with HbA1c in univariate analysis. This finding adds to other evidence suggesting that assessments of effortful control may help to predict which children are at greater risk of struggling with diabetes management [51]. However, child effortful control did not predict unique variance in HbA1c above and beyond parent marital relationship satisfaction. This suggests that parent marital relationship satisfaction might be a more primary and potent target for intervention. Longitudinal studies will be an important next step in research to disentangle the associations between child effortful control, parent marital satisfaction, and glycemic outcomes. However, intervention research points to parent marital relationship satisfaction, and not child effortful control, as a modifiable factor. That is, despite increasing research on psychosocial interventions for modifying child and adolescent effortful control, support for the efficacy of these approaches remains limited [52]. Yet, there is a robust evidence base for psychosocial interventions to support marital relationship quality [53], which might be translated into the type 1 diabetes context.

We also found significant associations of demographic factors with HbA1c. Higher GPA was associated with lower HbA1c in several of our analyses and this is consistent with previous research on academic achievement and glycemic outcomes [30, 54]. It is likely that the same family dynamics and parenting patterns that provide support for glycemic control similarly influence GPA. In addition, consistent with widely reported health disparities in glycemic outcomes [55–57], our findings reveal a strong trend toward higher HbA1c in children of color and Hispanic ethnicity after more than 2 years duration of type 1 diabetes. Our findings additionally showed an association between higher HbA1c and lower household income in univariate analysis, which is consistent with prior research [58, 59]. Last, in the bivariate associations, lower paternal education level was linked with higher HbA1c, which is consistent with research suggesting that both parental education and the father’s engagement and problem-solving ability in diabetes management are beneficial for children with type 1 diabetes [7, 60–62].

The current study has several limitations. Measures used to assess marital satisfaction and perception of conflict were not type 1 diabetes-specific. Additionally, information related to technology use and data obtained from technology (e.g. continuous glucose monitors) was not collected and could provide additional measures of glycemic control. Further, the relatively small sample size may have limited our ability to detect differences of smaller magnitude, including some that have been found in prior work, such as parent neuroticism. Due to the higher incidence of type 1 diabetes in white populations [63], the number of participants who were persons of color or of Hispanic ethnicity was small and these groups were therefore combined to allow analysis. This approach did not allow meaningful analyses of differences in glycemic control among specific racial/ethnic groups.

Additionally, families with married parents were excluded from the study if either parent stated that he/she was unwilling to complete the questionnaires. This exclusion criterion may have selected for families with higher marital relationship satisfaction and may have biased our results away from finding a significant effect by restricting the range of marital relationship satisfaction reports. As HbA1c was associated only with primary caregiver relationship satisfaction, this exclusion criterion could be eliminated from future studies possibly allowing for a wider range of responses. In the current study, we did not collect extensive parental demographic data (age, race/ethnicity, precise duration of the relationship), and this information could also be informative for future studies. Futher, while we included socioeconomic status indicators of income and education level, we did not collect data on insurance type and future research might further consider the insurance quality context in these associations. Finally, in this study, we found an association between low parental marital relationship satisfaction and less optimal glycemic outcomes, however, those data alone could suggest an alternative explanatory relationship whereby greater difficulty managing a child’s diabetes strains spousal relationships leading to poor relationship satisfaction. Data from a prospectively enrolled sample would therefore be important to understand the temporal process and confirm that low satisfaction with spousal relationships at diagnosis leads to less optimal future glycemic outcomes.

Although it is known that family dynamics affect glycemic control in children with type 1 diabetes, prior work has mainly focused on marital family structure or family conflict as opposed to non-structural factors such as the marital relationship. Our findings reveal that the primary caregiver’s involvement in a satisfying marital relationship is correlated with with glycemic outcomes. This is a novel finding which indicates that clinicians should consider exploring marital relationship satisfaction as part of their clinical care of children with type 1 diabetes. Clinicians might consider asking caregivers if they have a partner or person that helps support them with their child’s diabetes management tasks and how helpful that support is, in order to facilitate ongoing conversations with caregivers about their support system and its impact on diabetes management success.

## Data Availability

Data is available for review upon reasonable request.

## Code Availability

Available for review upon reasonable request.
